# Cyanobacteria: Photoautotrophic Microbial Factories for the Sustainable Synthesis of Industrial Products

**DOI:** 10.1155/2015/754934

**Published:** 2015-06-25

**Authors:** Nyok-Sean Lau, Minami Matsui, Amirul Al-Ashraf Abdullah

**Affiliations:** ^1^Centre for Chemical Biology, Universiti Sains Malaysia, 11900 Bayan Lepas, Penang, Malaysia; ^2^Synthetic Genomics Research Team, RIKEN Centre for Sustainable Resource Science, Biomass Engineering Research Division, Yokohama, Kanagawa 230-0045, Japan; ^3^School of Biological Sciences, Universiti Sains Malaysia, 11800 Penang, Malaysia

## Abstract

Cyanobacteria are widely distributed Gram-negative bacteria with a long evolutionary history and the only prokaryotes that perform plant-like oxygenic photosynthesis. Cyanobacteria possess several advantages as hosts for biotechnological applications, including simple growth requirements, ease of genetic manipulation, and attractive platforms for carbon neutral production process. The use of photosynthetic cyanobacteria to directly convert carbon dioxide to biofuels is an emerging area of interest. Equipped with the ability to degrade environmental pollutants and remove heavy metals, cyanobacteria are promising tools for bioremediation and wastewater treatment. Cyanobacteria are characterized by the ability to produce a spectrum of bioactive compounds with antibacterial, antifungal, antiviral, and antialgal properties that are of pharmaceutical and agricultural significance. Several strains of cyanobacteria are also sources of high-value chemicals, for example, pigments, vitamins, and enzymes. Recent advances in biotechnological approaches have facilitated researches directed towards maximizing the production of desired products in cyanobacteria and realizing the potential of these bacteria for various industrial applications. In this review, the potential of cyanobacteria as sources of energy, bioactive compounds, high-value chemicals, and tools for aquatic bioremediation and recent progress in engineering cyanobacteria for these bioindustrial applications are discussed.

## 1. Introduction

Cyanobacteria, also referred to as blue-green algae, are the oldest photosynthetic organisms on earth that originated approximately 2.6–3.5 billion years ago [1]. Indeed, the origin of photosynthetic organelle in eukaryotes is thought to have possibly arisen by the process of endosymbiosis between a phagotrophic host and a cyanobacterium [2]. Cyanobacteria are morphologically diverse and exist in different forms including unicellular, filamentous, planktonic or benthic, and colonial (coccoid) ones [3, 4]. They are by far the most widespread occurring photosynthetic organisms. They can thrive in a wide range of ecological habitats, ranging from marine, freshwater, to terrestrial environments. Cyanobacteria are also well known for their ability to perform different modes of metabolism and the capacity to switch rapidly from one mode to another [5]. All cyanobacteria are capable of oxygenic photosynthesis but some cyanobacterial species can switch to sulfide-dependent anoxygenic photosynthesis [6]. In dark or under anoxic conditions, cyanobacteria can perform fermentations for energy generation [7]. Some filamentous cyanobacteria have evolved specialized cells known as heterocysts to carry out nitrogen fixation [8].

At present, many bioindustrial processes rely on the fermentations of heterotrophic bacteria to produce various fine chemicals such as vitamins, enzymes, and amino acids. Nevertheless, the economic viability of these production schemes is limited by the cost of carbon substrates used in the fermentation processes. Cyanobacteria, endowed with photosynthesis system to fix carbon dioxide into reduced form, are ideal biosynthetic machinery for sustainable production of various chemicals and biofuels. Unlike heterotrophic bacteria, cyanobacteria require only sunlight, carbon dioxide, water, and minimal nutrients for growth, eliminating the cost of carbon sources and complex growth media. Sunlight is the most readily available and inexpensive resource on earth and the use of cyanobacteria for the production of fine chemicals and biofuels from solar energy offers a greener path for the synthesis process. Equipped with superior photosynthesis capabilities, cyanobacteria have higher photosynthesis and biomass production rates compared to plants and can convert up to 3–9% of the solar energy into biomass compared to ≤0.25–3% achieved by crops, for example, corn, sugar cane [9]. They also require less land area for cultivation than terrestrial plant, reducing the competition for arable land with crops intended for human consumption. Cyanobacteria utilize carbon dioxide, a type of greenhouse gases, during photosynthesis and help to achieve a carbon neutral production process. Being prokaryotes, cyanobacteria possess relatively simple genetic background that eases manipulation [10]. In addition, the residual cyanobacteria biomasses that are left over after high-value products extraction can be used as animal feed or converted into organic fertilizer.

Considering the aforementioned inherent merits of cyanobacteria, they are one of the attractive candidates for use in diverse biotechnological application. With the recent advances in genetic and metabolic engineering technologies and the availability of more than 300 cyanobacterial genome sequences, there is significant progress in research directed towards realizing the full potential of these photosynthetic bacteria. Cyanobacteria have gained considerable attention in recent years for their possible use in agriculture, nutraceuticals, effluent treatment, and the production of biofuels, various secondary metabolites including vitamins, toxins, and enzymes. In this paper, the recent progress in developing cyanobacteria for various potential applications in biotechnology is discussed.

## 2. Potential Applications of Cyanobacteria as Energy Sources

Anticipation of depletion of fossil fuel resources and global warming have spurred vigorous research initiatives aimed at developing carbon neutral alternatives to supplement or replace fossil fuels. Several alternatives to current fossil fuels have been proposed and they include ethanol, 1-butanol, isobutanol, hydrogen gas, and alkanes.

**Figure 1:**
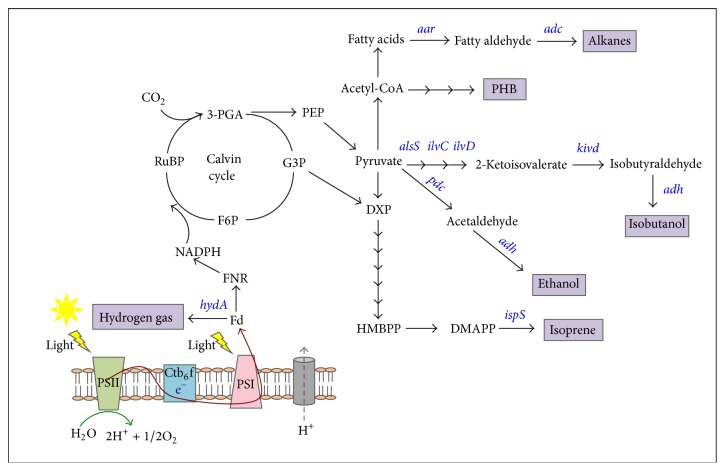
A schematic representation of biochemical pathways for various industrial products synthesis in cyanobacteria. 3-PGA, 3-phosphoglycerate;* aar*, aldehyde decarbonylase;* adc*, alcohol dehydrogenase;* alsS*, acetolactate synthase; F6P, fructose-6-phosphate; FNR, ferredoxin NADP^+^ reductase; G6P, glucose-6-phosphate; HydA, [FeFe] hydrogenase;* ilvD*, dihydroxy-acid dehydratase;* ilvC*, acetohydroxy acid isomeroreductase;* pdc*, pyruvate decarboxylase; PEP, phosphoenolpyruvate; PHB, polyhydroxybutyrate.

The production of ethanol via biological route has received widespread attention in recent years. Traditionally a two-step route to first collect plant-derived biomass and subsequent conversion of the biomass to fuels by microbial fermentation is employed [30]. This indirect production scheme is inefficient in the conversion of biomass to fuels [31] and thus there are increasing interests in the use of photosynthetic microbes to directly convert carbon dioxide to fuels. Although some cyanobacterial strains naturally produce low level of ethanol as a byproduct of natural fermentation, it is necessary to enhance the production efficiency of cyanobacteria to reach an economically viable level. An attempt to introduce the pyruvate decarboxylase (*pdc*) and alcohol dehydrogenase II (*adh*) genes from* Zymomonas mobilis* into the chromosome of* Synechocystis* sp. PCC 6803 was reported [11] (Figure 1). Photosynthetic production of up to 550 mg/L ethanol was achieved in the engineered* Synechocystis* sp. [32]. Further engineering of* Synechocystis* sp. by overexpressing endogenous alcohol dehydrogenase and disrupting polyhydroxyalkanoate biosynthetic pathway increased ethanol production up to 5500 mg/L [12] (Table 1). Isobutanol and 1-butanol are considered as better substitutes for gasoline compared to ethanol as they have greater energy density, being less corrosive and less volatile [33]. Using a similar genetic modification approach, direct photosynthetic production of isobutanol in cyanobacteria is also feasible. The introduction of an artificial isobutanol biosynthesis pathway into* Synechococcus elongatus* PCC 7942 had resulted in the production of isobutyraldehyde and isobutanol up to 1100 and 450 mg/L, respectively [13].

More than 14 genera of cyanobacteria are known to produce hydrogen under various culture conditions and they include* Anabaena*,* Aphanocapsa*,* Calothrix*,* Microcystis*,* Nostoc*, and* Oscillatoria* [15, 34–36]. In heterocystous or filamentous cyanobacteria, hydrogen gas is produced as a byproduct of nitrogen fixation under nitrogen limiting growth conditions. In cyanobacteria, the production of hydrogen gas is also facilitated by the reversible activity of hydrogenases enzymes. Two distinct types of hydrogenases are present in different cyanobacterial species: uptake hydrogenases that oxidize oxygen; bidirectional or reversible hydrogenases that can both take up or produce hydrogen [37]. The efficiency of hydrogen production in some cyanobacteria is limited by the extreme oxygen sensitivity of hydrogenases and the tendency for [NiFe] hydrogenases to thermodynamically favor hydrogen uptake [38]. Increased production of hydrogen is achievable by blocking pathways that compete for reductant consumption with hydrogenases. The disruption of* ldhA* gene that is responsible for NADH consumption in lactate production in* Synechococcus* sp. PCC 7002 had resulted in a significant increase in the NADH/NAD^+^ ratio and a concomitant fivefold increase in hydrogen production by the native bidirectional [NiFe] hydrogenase [39]. In another example, enhanced hydrogen production in* S*.* elongatus* PCC 7942 was obtained through heterologous expression of exogenous [FeFe] hydrogenases (HydA) from* Clostridium acetobutylicum* [16]. The expression of [FeFe] hydrogenases that thermodynamically favor hydrogen production relative to [NiFe] hydrogenase can modulate redox flux in the heterologous host, resulting in higher hydrogen production. Metabolic engineering of cyanobacteria by redirecting glycogen catabolism through the oxidative pentose pathway was found to enhance intracellular NADPH concentrations and consequently improve the hydrogen yield. In the glyceraldehyde-3-phosphate (*gap*1) gene deletion and NAD^+^-glyceraldehyde-3-phosphate (GAPDH-1) overexpression strains of* Synechococcus* sp. PCC 7002, 2.3-fold and 3-fold increase in hydrogen production, respectively, were obtained compared to the wild type [40].

Some cyanobacteria are known to synthesize alkanes or alkenes that have desirable properties for combustion. Studies on the alkane biosynthetic pathways in cyanobacteria revealed that the two important enzymes acyl-acyl carrier protein reductase and aldehyde decarbonylase are responsible for the conversion of fatty acid metabolism intermediates to alkanes or alkenes [41, 42]. Heterologous expression of genes encoding these two enzymes had conferred the ability to produce and secrete alkane in* Escherichia coli* [43]. In addition, the expression of acyl-acyl carrier protein reductase and aldehyde decarboxylase genes from* S*.* elongatus* PCC 7942 in* Synechococcus* sp. PCC 7002 was found to increase alkane production in the heterologous host [17]. In another example, the overexpression of both acyl-acyl carrier protein reductase and aldehyde-deformylating oxygenase from several cyanobacterial strains was shown to increase alkanes/alkenes yield by twofold [44]. The production of various biofuel precursors including alkanes, fatty acids, and wax esters from fatty aldehydes was achievable in* Synechocystis* sp. PCC 6803 through pathway engineering. Overexpression of acyl-ACP reductase, the enzyme that converts the end product of fatty acid biosynthesis into acyl aldehyde, resulted in a significant improvement in fatty acids production in* Synechocystis* sp., in addition to alkane production [45]. In another study, the coproduction of alkanes and *α*-olefins was observed in* Synechococcus* sp. NKBG15041c by expressing the acyl-acyl carrier protein reductase/aldehyde-deformylating oxygenase pathway genes from* S*.* elongatus* PCC 7942 [46].

## 3. Role of Cyanobacteria in Aquatic Bioremediation

In recent years, biological waste treatment systems, in particular the use of cyanobacteria in wastewater treatment, have attracted considerable scientific and technical interest. Cyanobacteria are characterized by the ability to oxidize oil components, complex organic compounds, and accumulate metal ions, for example, Zn, Co, and Cu [47]. Thus, cyanobacteria are promising tool for the secondary treatment of urban, agricultural, or industrial effluents (Table 2).

The potentials of cyanobacteria in the removal of nutrients from wastewater rich in nitrogenous and phosphorus compounds have been demonstrated. The ability to remove nitrogenous and phosphate ions from wastewater was observed in cyanobacteria such as* Oscillatoria*,* Phormidium*,* Aphanocapsa*, and* Westiellopsis* [19–21, 48]. The biomass of* Spirulina* strains contains different functional groups, for example, carboxyl, hydroxyl, sulfate, and other charged groups that are important for metal binding. They have great potentials in metal pollution control and the biosorption of zinc and nickel by several strains of* Spirulina*, namely,* Spirulina indica*,* Spirulina maxima*, and* Spirulina platensis*, was investigated recently [22]. Pesticide application is known to have negative impacts on soil ecology and cyanobacteria have been reported to accumulate and detoxify these pesticides. Previous studies demonstrated that* Synechocystis sp.* PUPCCC 64,* Westiellopsis prolifica, Nostoc hatei*, and* Anabaena sphaerica* are able to degrade organophosphorus or organochlorine insecticides in the aquatic environment [26, 27].

One of the drawbacks that limit the practical applications of cyanobacteria in wastewater treatment is the difficulty in the separation of biomass from the effluent before discharge [49]. The use of immobilization to entrap cyanobacteria in matrices (agarose, carrageenan, chitson, alginate, and polyurethane foam) can help to solve the harvesting problem. In addition, immobilized cyanobacteria show higher efficiency in nutrient or metal removal compared to their free-living counterpart. Immobilization of* Anabaena doliolum* was shown to increase its copper and iron ions removal capacity in the order of 45 and 23% higher than its free-living counterpart [23]. Similarly, the efficiency to take up nitrogenous and phosphorus compounds was enhanced in immobilized* Chlorella* and* Anabaena* [23].

In natural environment, many cyanobacteria form symbiotic associations with other aerobic or anaerobic microorganisms. It is interesting to note that cyanobacterial mats including* Oscillatoria*,* Synechocystis*, and* Pleurocapsa* have been shown to aid in the degradation of hydrocarbons present in oil. Although cyanobacteria are not directly responsible for the degradation of hydrocarbons, they facilitated the degradation process by providing oxygen and nutrients to the associated oil-degrading bacteria [50]. A consortium comprising* Phormidium*,* Oscillatoria*,* Chroococcus*, and the oil-degrading bacterium,* Burkholderia cepacia*, was successfully developed and employed on a rotating biological contactor to efficiently degrade petroleum compounds [51].

## 4. Cyanobacteria as Potential Bioactive Compounds Sources

Cyanobacteria have been identified as sources of bioactive compounds with interesting biological activities, for example, antibacterial, antifungal, antiviral, antialgal, anticancer, anti-inflammatory, and so forth. These bioactive compounds include lipopeptides (40%), amino acids (5.6%), fatty acids (4.2%), macrolides (4.2%), and amides (9%) [4]. The excretion of bioactive compounds by cyanobacteria into the aquatic environments is possible allelopathy strategy used by cyanobacteria to outcompete other microorganisms within the same ecosystem [52]. These allelopathic compounds include alkaloids, cyclic peptides, terpenes, and volatile organic compounds. Cyanobacterial allelochemicals are found to exhibit inhibitory effects toward the growth, photosynthesis, respiration, carbon uptake, and enzymatic activity of algae as well as induce oxidative stress in algae [53]. To date, several allelochemicals from cyanobacteria have been identified and they include cyanobacterin produced by* Scytonema hofmanni*, enediyne-containing photosystem II inhibitor synthesized by* Fischerella muscicola*,* hapalindoles* that are isolated from* Hapalosiphon* and* Fischerella* spp., and nostocyclamides from* Nostoc* sp. These antialgal compounds synthesized by cyanobacteria can be developed as alternative to synthetic algicides that are used to control harmful algal blooms.

Extensive screenings are presently undergoing to search for new antibacterial compounds from cyanobacterial extracts that can be potentially developed as new drugs or antibiotics. It has been reported that cyanobacterial extracts of* Westiellopsis prolifica* ARM 365,* Hapalosiphon hibernicus* ARM 178,* Nostoc muscorum* ARM 221,* Fischerella* sp. ARM 354, and* Scytonema* sp. showed antibacterial activity against* Pseudomonas striata, Bacillus subtilis, E*.* coli*, and* Bradyrhizobium* sp. [54]. In another study,* Anabaena* sp. was shown to exhibit antibacterial activity against* Staphylococcus aureus*,* E*.* coli*,* Pseudomonas aeruginosa*,* Salmonella typhi*, and* Klebsiella pneumonia* [55]. Noscomin, a diterpenoid compound isolated from* Nostoc commune*, showed antibacterial activity against* Bacillus cereus*,* Staphylococcus epidermidis*, and* E. coli* at MIC values comparable with those obtained for standard drugs [56]. Additionally, cyanobacteria produce a broad spectrum of compounds with antifungal activity. For example, nostofungicide isolated from* N*.* commune* is effective against* Aspergillus candidus*; norharmane from* Nostoc insulare* and 4,4′-dihydroxybiphenyl from* Nodularia harveyana* showed potent antifungal activity against* Candida albicans* [57, 58]. Antiviral activity of extracts or compounds isolated from cyanobacteria has also been reported. Cyanovirin-N, scytovirin N, and sulfoglycolipid isolated from* Nostoc ellipsosporum*,* Scytonema varium*, and* Scytonema* sp., respectively, are shown to exhibit potent antiviral activity against human immunodeficiency virus (HIV) [59–61].

## 5. Cyanobacteria as Sources of Value-Added Products

### 5.1. Pigments, Vitamins, and Enzymes

Cyanobacteria are known to produce various fine chemicals and there are considerable interests in the production of these chemicals from cyanobacteria on a commercially viable scale. Two important cyanobacterial pigments, phycobiliproteins and carotenoids, are extensively used in bioindustry and have high commercial value. Phycobilisomes are the major light-harvesting complexes present in cyanobacteria and they consist mainly of phycobiliproteins such as phycocyanin, allophycocyanin, and phycoerythrin. On the other hand, the major carotenoids accumulated by cyanobacteria are beta-carotene, zeaxanthin, nostoxanthin, echinenone, and canthaxanthin. These pigments are commonly used as food colorants, food additives, and supplements for human and animal feeds. Carotenoids are well known for their antioxidant properties and their possible role in the prevention and control of human health and disease conditions, for example, cancer, cardiovascular problems, cataracts, and muscular dystrophy, has been reported [62].

Some marine cyanobacteria are valuable sources of vitamins and they are being used for the large-scale production of vitamins of commercial interest such as vitamins B and E. For example,* Spirulina* (*Arthrospira*) is known to be a rich source of vitamin B12, beta-carotene, thiamine, and riboflavin. It is marketed in the form of powder, granules, tablets, or capsules and commercially available* Spirulina* tablets contain up to 244 *μ*g of vitamin B12 per dry weight [63]. Cyanobacteria are found to secrete a broad spectrum of enzymes that can be exploited for commercial applications. These industrially important enzymes include protease, amylase, and phosphatases. Proteases are predominantly used for food processing industries; alpha-amylases are extensively used in starch industries and phosphatases and acid phosphatases are widely used as diagnostic markers [64].

### 5.2. Isoprene

Isoprene is an energy rich hydrocarbon that is potentially a biofuel and an important feedstock in the synthetic chemistry industry. It is used industrially as the starting material to make synthetic rubber. Although isoprene is naturally synthesized and released by many herbaceous, deciduous, and conifer plants into the surrounding environments, it is impractical to harvest isoprene from plant in large-scale. The production of isoprene by photosynthetic cyanobacteria through heterologous expression of isoprene synthase (*IspS*) from* Pueraria montana* was demonstrated [65]. The accumulation of ~50 *μ*g isoprene per g dry cell weight per day was achievable in recombinant* Synechocystis* sp. PCC 6803 transformed with codon optimized* IspS* gene. In recent work, the introduction of isoprene synthase together with the mevalonic acid pathway genes was found to increase photosynthetic isoprene production yield to approximately 2.5-fold compared with cyanobacteria transformed with* IspS* gene only [66].

### 5.3. Biopolymers

Polyhydroxyalkanoate (PHA) is a type of biodegradable polymer that can serve as substitute for petroleum-based plastics and a biocompatible material that has promising applications in biomedical or pharmaceutical field. Several cyanobacteria including* Aphanothece* sp. [67],* Oscillatoria limosa* [68], some species of the genus* Spirulina* [69, 70], and the thermophilic strain* Synechococcus* sp. MA19 [71] are natural producers of PHA. However, the PHA yield obtained from direct photosynthetic production in cyanobacteria is low (<10%). Nutrient-limiting culture conditions and carbon feedstocks addition were found to enhance PHA accumulation. The applications of phosphorus-deficiency, gas-exchange limitation culture conditions as well as fructose and acetate addition had resulted in remarkable increases in PHA accumulation up to 38% of the dry cell weight in* Synechocystis* sp. PCC 6803 [72]. In another attempt to enhance photosynthetic PHA production in* Synechocystis* sp. PCC 6803, the introduction of an acetoacetyl-CoA synthase that catalyzes the irreversible condensation of acetyl-CoA and malonyl-CoA to acetoacetyl-CoA was found to have a positive impact on PHA production [73]. The highest PHA production obtained from photosynthetic cultures of genetically* Synechocystis* sp. was 14 wt%. High PHA accumulation of up to 52% dry cell weight was demonstrated in marine cyanobacterium* Synechococcus* sp. PCC 7002. The* Synechococcus* sp. was transformed with plasmid carrying* Cupriavidus necator* PHA biosynthetic genes using* recA* complementation as selection pressure for plasmid stability [74]. Interestingly, the ability of (*S*)- and (*R*)-3-hydroxybutyrate molecules synthesis and secretion from genetically engineered* Synechocystis* sp. PCC 6803 cells was also reported [75].

## 6. Challenges and Outlook

Considering the great potentials of cyanobacteria in diverse biotechnological applications, it would be of scientific, technological, and industrial interests to translate theses photosynthetic biological systems' potential into realizable large-scale production. Although a broad spectrum of natural products synthesized by cyanobacteria has potential bioindustrial applications, the underlying challenge in commercialization is to achieve production yields that meet realistic scalable production.

The potential uses of cyanobacteria as hosts for the production of various carbon-containing industrial products, for example, ethanol, isobutanol, hydrogen gas, and alkanes, have been extensively explored for the past ten years [13, 16, 17, 32, 33, 39]. In this production model, the cyanobacterial cells can be seen as photosynthetic microbial cell factories that harvest solar energy to fix carbon dioxide into products of interest. As the carbon content of the synthesized compound originates from the carbon dioxide fixed during photosynthesis, the productivity of the system depends on the efficiency of carbon dioxide fixation. On the other hand, photosynthetic efficiencies are affected by the efficiency of solar photon capture and the conversion of captured photon to chemically stored energy [33]. Although cyanobacteria are equipped with photosynthetic machineries that are two- to threefold more efficient in solar energy conversion than that of crop plants, the efficiencies are still low with yield less than 10% [9]. Genetic engineering approaches aiming at improving the photosynthetic efficiencies of cyanobacteria that will ultimately result in higher final product yield are being explored. RuBisCo is an essential enzyme that catalyzes the first major step of carbon fixation. However, it is a notoriously inefficient enzyme that has slow catalytic rate and is subject to competitive inhibition by O_2_. RuBisCo is suggested to be the rate-limiting enzyme in carbon fixation and a potential target for genetic manipulation to improve photosynthetic efficiency [76]. Overexpression of* Synechococcus* sp. PCC 6301 RuBisCo operon in* S*.* elongatus* PCC 7942 was found to enhance CO_2_ fixation and boost isobutanol production levels by approximately twofold in the genetically engineered strain [77].

Light availability plays pivotal role in determining the growth of photoautotrophs; thus, selective pressure has favored adaptations that maximize light capture in photosynthetic biological systems to compete for sunlight. However, under high light conditions, the excessive capture of photons by cells in the surface layer of cyanobacterial cultures is lost through thermal dissipation, limiting the availability of light to cells underneath. To address this issue, attempt to artificially reduce the light-harvesting antenna size was carried out. This strategy is designed to enable cells at the culture surface to capture only the amount of light that they require and allow greater penetration of excess light into the culture. In a phycocyanin-deficient* Synechocystis* strain, a substantial improvement in photosynthetic activity and biomass production was obtained relative to their wild-type counterparts [78]. As oxygenic photosynthesis in cyanobacteria effectively utilizes photons in the waveband of 400–700 nm, the remainder of the energy is lost as heat. In addition, the photosystems in most photosynthetic organisms compete for solar radiation with the same wavelengths, reducing the overall energy conversion efficiency. One of the interesting strategies to increase photosynthetic efficiencies suggests the engineering of one of the two photosystems to extend the absorption maxima to ~1100 nm [79].

While biotechnological production potentials of cyanobacteria have attracted considerable interest, high yield synthesis of industrial products from cyanobacteria remains challenging. In biofuels synthesis, the production ability of cyanobacteria is likely limited by the low level of cells tolerance to biofuel. RNA-sequencing was carried out to determine the transcriptome profile of* Synechocystis* sp. PCC6803 cultivated under different concentrations of exogenous* n*-butanol. The study revealed that the overexpression of several candidate proteins, particularly the small heat shock protein, HspA, improved the tolerance of* Synechocystis* sp. to butanol [80]. To understand the metabolomics changes related to butanol stress, metabolomics profile of* Synechocystis* sp. PCC 6803 cultivated under gradual increase of butanol concentration was determined. The identification of metabolites that were differentially regulated during the evolution process provides metabolomics basis for further engineering of cyanobacteria to improve their product tolerance [81].

Although most strategies implemented to engineer cyanobacteria as microbial cell factories for various high-value products synthesis focus on local pathway optimization, system biology approaches (e.g., transcriptomes, proteomics, and metabolomics) would enhance our understanding of cyanobacterial biochemistry. For example, recent transcriptomes studies on genetically engineered* Synechocystis* sp. PCC 6803 provide insights into understanding the effects of overexpressing PHA biosynthetic genes on photoautotrophic biopolymer production [73]. Constraint-based models of metabolism can be used to evaluate the effects of genetic manipulation or environmental perturbations on biomass yield and metabolic flux distribution. Based on mathematical models, the optimal intracellular metabolic flux profile to maximize the value of the selected objective function can be predicted. This* in silico* modeling approach provides a mean to design an optimal metabolic network to maximize the synthesis of any product of interest. A comprehensive genome-scale metabolic model for* Synechocystis* sp. PCC 6803 was reconstructed and analyzed to understand the photosynthetic process in cyanobacteria in mechanistic detail. Interestingly, the metabolic model revealed that the regulation of photosynthetic activity in* Synechocystis* sp. is rather complex and a high degree of cooperativity between nine alternative electron flow pathways is important for optimal photoautotrophic metabolism [82].

## 7. Conclusion

Owing to their simple growth requirements, ease of genetic manipulation, and ability to capture solar energy and fix atmospheric carbon dioxide directly into industrial products, the potentials of cyanobacteria for various biotechnological applications have been well recognized. However, the application of cyanobacterial cultures for large-scale synthesis of products of interest is technologically challenging. Efficient and cost-effective photosynthetic bioreactors need to be developed to achieve maximum productivity in large-scale cyanobacterial cultures with minimum operation costs. With the advances in genetic and metabolic engineering approaches as well as development of suitable cultivation systems, we can harness the photosynthetic efficiency of cyanobacteria to provide green paths for the synthesis of industrial products.

## Figures and Tables

**Table 1:** Biofuels production in cyanobacteria.

Cyanobacteria	Compound	Production	References
*Synechococcus elongatus* PCC 7942	Ethanol	230 mg/L	[11]
*Synechocystis* sp. PCC 6803	Ethanol	5500 mg/L	[12]
*Synechococcus elongatus* PCC 7942	Isobutanol	450 mg/L	[13]
*Synechococcus elongatus* PCC 7942	Isobutyraldehyde	1100 mg/L	[13]
*Synechococcus elongatus* PCC 7942	1-Butanol	29.9 mg/L	[14]
*Anabaena* sp. PCC 7120	Hydrogen	2.6 *μ*mol mg^−1^ chl *a* h^−1^	[15]
*Anabaena cylindrica* IAM M-1	Hydrogen	2.1 *μ*mol mg^−1^ chl *a* h^−1^	[15]
*Nostoc commune* IAM M-13	Hydrogen	0.25 *μ*mol mg^−1^ chl *a* h^−1^	[15]
*Synechococcus elongatus* PCC 7942	Hydrogen	2.8 *μ*mol mg^−1^ chl *a* h^−1^	[16]
*Synechococcus* sp. PCC 7002	n-Alkanes	5% dry cell weight	[17]
*Synechocystis* sp. PCC 6903	Fatty acids	197 mg/L	[18]

**Table 2:** Potential uses of cyanobacteria in bioremediation of wastewater.

Cyanobacteria	Types of wastewater	Compounds removed	References
*Phormidium bohneri *	Industrial effluent (cheese factory)	NO_3_^−^ and PO_4_^3−^	[19]

*Phormidium bohneri *	Swine manure effluent	NH_4_^+^ and PO_4_^3−^	[20]

*Oscillatoria* sp.	Activated sludge effluent	NO_3_^−^ and PO_4_^3−^	[21]

*Schizothrix calcicola*, *Phormidium subfuscum*, *Phormidium tenue*, and *Oscillatoria* sp.	Synthetic wastewater	NO_3_^−^ and PO_4_^3−^	

*Spirulina indica*,* Spirulina maxima*, and *Spirulina platensis *	Synthetic heavy metal solution	Nickel and zinc	[22]

*Anabaena doliolum*, *Chlorella vulgaris *	Synthetic wastewater	Copper, iron, NH_4_^+^, and NO_3_^−^	[23]

*Nostoc* sp. PCC 7936	Industrial wastewater [chromium (VI) plating industry]	Chromium (VI)	[24]

*Anabaena oryzae*, *Anabaena variabilis*, and *Tolypothrix ceytonica *	Mixed domestic-industrial wastewater	Organic matter, copper, and zinc	[25]

*Synechocystis* sp. PUPCCC 64	Synthetic insecticide solution	Chlorpyrifos	[26]

*Westiellopsis prolifica*, *Nostoc hatei*, and *Anabaena sphaerica *	Synthetic insecticide solution	Carbofuran, chlorpyrifos, and endosulfan	[27]

*Synechocystis* sp. PUPCCC 64	Synthetic herbicide solution	Anilofos	[28]

*Anabaena oryzae*, *Nostoc muscorum*, and *Spirulina platensis *	Synthetic pesticide solution	Malathion	[29]
